# Molecular functions of metallothionein and its role in hematological malignancies

**DOI:** 10.1186/1756-8722-5-41

**Published:** 2012-07-27

**Authors:** Shinichiro Takahashi

**Affiliations:** 1 Division of Hematology, Kitasato University School of Allied Health Sciences, 1-15-1 Kitasato, Minami-ku, Sagamihara, 252-0373, Japan; 2 Division of Molecular Hematology, Kitasato University Graduate School of Medical Sciences, 1-15-1 Kitasato, Minami-ku, Sagamihara, 252-0373, Japan

**Keywords:** Metallothionein (MT), Molecular function, Hematological malignancies

## Abstract

Metallothionein (MT) was reported to be a potential negative regulator of apoptosis, and various reports have suggested that it may play roles in carcinogenesis and drug resistance, in at least a portion of cancer cells. The author summarizes the current understanding of the molecular functions of MT for tumor cell growth and drug resistance. These activities are regulated through intracellular metal ion modulation and free radical scavenging. Compared with analyses of solid tumors, few studies have analyzed the roles of MT in hematological malignancies. This review mainly describes the functions of MT in hematopoietic cells. Furthermore, through expression analyses of leukemias and lymphomas, the roles of MT in the biology of these diseases are particularly focused upon.

## Introduction

Metallothionein (MT) was first isolated by Margoshes and Vallee [1] as a cadmium-binding low-molecular weight protein from the horse kidney. MT proteins comprise a group of low-molecular weight cysteine-rich intracellular proteins [2]. Structural studies have shown that these unusual proteins with 61 amino acids can bind to both essential metals (zinc and copper) and toxic metals (cadmium and mercury). MTs are characterized by their low molecular weight, high cysteine content, lack of aromatic amino acid residues and presence of 7–12 metal atoms per molecule [3,4]. Owing to their rich thiol content, MTs bind a number of trace metals including zinc, cadmium, mercury, platinum and silver, and also protect cells and tissues against heavy metal toxicity. The level of metal ions, affects on normal hematopoietic cell proliferation and differentiation. For example, zinc deficiency in mice result in the overall decline in the absolute number of lymphocytes in concordance with increment of the granulocytes and monocytes [5]. Since MT binds to metal ions, MTs may play a role in the hematopoietic cell proliferation/differentiation.

Two major isoforms of MT, designated MT-I and MT-II, that can be separated by ion-exchange chromatography have been identified in mammals, and are found in all types of tissues [6,7]. Moreover, two other members, designated MT-III and MT-IV, are expressed in limited tissues as minor isoforms [8,9]. In humans, the MT genes are located on chromosome 16 q13 in a cluster and may involve at least 10 identified functional genes [2]. Although the MT-II, MT-III and MT-IV proteins are encoded by a single gene, the MT-I protein comprises many subtypes encoded by a set of MT-I genes. The known functional MT-I and MT-II isoforms are MT-IA, -IB, -IE, -1F, -IG, -IH, -IX and –IIA [2]. Differential regulation in response to heavy metals has been reported for some members of MT gene family. For example, quantitative analysis of MT-IIA, MT-IF and MT-IG gene expression in HepG2 cell line has indicated that these genes are differentially regulated in terms of both the rate and extent of transcript accumulation [10]. The MT-IIA and MT-IG mRNA levels are approximately 20- and 4-fold higher than that of MT-IF mRNA, respectively. The expression of the MT-IIA and MT-IA genes have been detected in all cell types studied [11,12], while other MT genes such as MT-IB and MT-IE are expressed in a cell-type specific manner [13,14]. Different cells express different MT isoforms with varying levels of expression perhaps as a result of the different function of each isoform. Therefore, the fact that multiple MT isoforms exist, and the great variety of substances and agents that acts as inducers, complicates the search for the biological role of each MT isoform.

To date, there are no studies for mice with single MT-I or -II deletion, therefore, the functional difference between MT-I and II remain obscure. However, several groups have reported phenotype of double MT-I and -II gene knockout mice [15,16]. From these studies, although some differences in phenotype and metabolic responses may exist, susceptibility to heavy metal toxicity, response to inflammation and zinc homeostasis is common features of the phenotypes of these double knockout mice.

Transgenic mice with multiple MT-I genes, have 10–20 fold greater MT protein levels in the pancreas, liver and stomach, and 2–6 fold greater MT levels in a number of other organs including kidney, spleen and heart [17]. They have 50% more zinc in liver and 300% more in the pancreas [17]. Pancreatic MT of these mice has been found to be a very sensitive indicator of zinc status [18].

A number of studies have shown that increased MT expression is closely associated with tumor grade and proliferative activity in solid tumors [2]. Another study reported increased levels of MT-I and MT-II mRNA and protein expression in various human cancers, such as breast, kidney, lung, nasopharyngeal, ovarian, prostate, salivary gland, testicular, urinary bladder, cervical and endometrial cancers, as well as skin carcinoma and melanoma [19]. However, MT-I and MT-II are downregulated in other types of tumors, e.g. hepatocellular, gastric, colorectal, central nervous system and thyroid cancers. Several lines of evidence suggest roles for MTs in cancer development, treatment resistance and poor prognosis [20,21]. Studies of the mutations of MT using clinical specimens that could influence the expression of this gene are scarce. Tai *et al.*[22] demonstrated nonsilent mutations in MT-IH gene in several breast cancer cell lines, however, there are no differences of MT-IH expression in tumors and normal tissues.

Several reports have suggested that MTs are also involved in the pathogenesis of hematological malignancies [23-29]. Furthermore, the author’s group recently demonstrated that MT-I expression is directly regulated by the hematopoietic transcription factor PU.1 [26]. Overexpression of MT-I was significantly correlated with downregulation of PU.1, which is frequently observed in various hematological malignancies such as acute myeloid leukemia (AML) [26]. However, compared with other tumors, studies of MT in hematological malignancies are relatively scarce. The aim of this review is to describe the roles of MT in hematological malignancies. Furthermore, this review summarizes the current understanding of the molecular functions of MT.

### MT in cell growth

**Figure 1:**
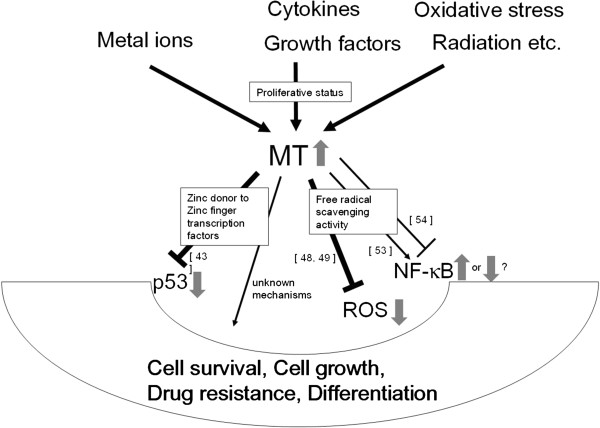
**Schematic presentation of the stimuli that induce MT, and the downstream effects of MT overexpression.** Two potential mechanisms that facilitate tumor cell growth or drug resistance are illustrated as downstream effects of MT overexpression. The indicated numbers are the references cited in this review.

MT can be activated by a variety of stimuli, including metal ions, cytokines and growth factors [2]. In addition, MT was reported to be induced by radiation [30]. In fact, the synthesis of MT was shown to be increased by several-fold during oxidative stress [31] to protect the cells against cytotoxicity [32] and DNA damage [33]. The stimuli that induce MT and the downstream effects of MT overexpression are summarized in Figure 1. MT expression in tumor tissues is mainly correlated with the proliferative capacity of tumor cells [34]. However, there are few exceptional cases like downregulation of MT-I, -II in hepatocellular carcinoma [35], and also reduced level of intracellular zinc result in the increment of granulocyte, but the decreased number of lymphocytes [5].

MT regulation during cell cycle progression has been demonstrated in normally cycling cells. Maximal nuclear accretion of MT by two- to three-fold the basal level was found to coincide with the S and G_2_ phases, while high cytoplasmic expression occurred during late G_1_ phase and G_1_/S transition and basal amounts were found in G_0_ phase [36,37]. The MT-I location on the antiapoptotic function was studied by Levadoux-Martin *et al.*[38]. They revealed that the perinuclear localization of MT-I mRNA is important for the function of MT in a protective role against DNA damage and apoptosis induced by external stress. In another study, four-fold increases in MT-II were found in proliferating liver cells compared with those in growth arrest, and there was evidence for translational control as well as a slower rate of MT-II degradation in proliferating cells [39].

MT can facilitate tumor cell growth or drug resistance by two potential mechanisms (Figure 1). First, MT may play an essential role in development and growth through the maintenance of zinc metabolism. The translocation of MT into the nucleus during the proliferative phase (G_1_–S) of the cell cycle in human tumors also supports a zinc donor role for MT during tumor growth [40]. The nuclear accumulation of MT may be important for supplying zinc or other metals to target molecules, including enzymes, zinc-finger transcription factors and tumor suppressor gene products such as p53 [6,41-45]. Meplan *et al.*[43] demonstrated that zinc incorporation is required for the stablization of wild-type recombinant p53 in a form capable of binding speficically to DNA. They also showed that human recombinant thionein, the metal-free form of MT, reported to remove zinc from zinc finger transcription factors Sp1 thereby abrogating their transcriptional activity [46,47], inhibited binding of p53 to a specific consensus sequence *in vitro*. Supplementation of thionein with equimolar amounts of zinc prior to incubation with p53 abrogated this effect. Co-transfection experiment using p53-dependent reporter gene with p53 and MT expression vectors revealed that, when transfected in excess of MT as compared to p53, MT exerts an inhibitory effect, by an average of 50%, consistent with a metal chelator effect of MT. However, when expressed at lower levels, MT may catalyze metal-transfer reactions regulating the folding of the DNA-binding domain of p53, thus enhancing its activity of p53 to approximately 300% [43]. Second, MT can protect the cells against radiation and chemotherapeutic agents by virtue of its free radical scavenging property [48,49]. The antioxidant properties of MT contribute to its antiapoptotic function [50]. In particular, MT synthesis is induced following treatment with cadmium, an environmental pollutant that causes oxidative stress, DNA damage and apoptosis [51,52]. Nuclear factor (NF)-κB is a transcription factor that also plays a role in the antiapoptotic function of MT. NF-κB activity is regulated by the intracellular redox status, and reactive oxygen species may be involved in the NF-κB activation cascade. Abdel-Mageed *et al.*[53] revealed that, in breast carcinoma MCF-7 cells, MT caused transactivation of NF-κB through a direct interaction. They suggested a potential role for NF-κB in mediating the antiapoptotic effects of MT. However, Sakurai *et al*. [54] demonstrated that the DNA-binding activity of NF-κB is impaired in MT null embryonic cell lines. Although the role of MT for NF-κB activation remains controversial, these findings indicate that MT expression may be necessary for the growth and survival of tumor cells.

### MT in drug resistance

The protective roles of MT against oxidative stress and metal toxicity suggest that MT may also have a functional role in drug resistance. Using several cell lines (PLC/PRF/5, H460 and HepG2), Shimoda *et al.*[55] revealed that induced MT levels were negatively correlated with sensitivity to etoposide-induced apoptosis, suggesting that MT is a potential negative regulator of apoptosis. Kondo *et al.*[56] investigated embryonic fibroblast cells from transgenic mice with targeted disruptions of the MT-I and MT-II genes (MT^−/−^), and found that MT^−/−^ cells showed enhanced sensitivity to a 2-h exposure to anticancer drugs, including cisplatin, melphalan, bleomycin and cytarabine, compared with wild-type cells (MT^+/+^). In addition, both human primary hepatocellular carcinoma and metastatic carcinoma had low MT levels and higher numbers of apoptotic cells compared with the normal liver [57,58].

However, the role of MT in the development of chemoresistance in the clinical setting is still controversial. Studies in human tumors, such as ovarian, testicular and colon tumors, appear to suggest that overexpression of MT may have protective effects against antineoplastic agents [59,60], whereas other reports do not support this perception [61,62].

### MT functions in hematopoietic cells

Mouse embryonic stem (ES) cells, remain pluripotent in vitro when grown in the presence of leukemia inhibitory factor (LIF). However, it was shown that apoptosis but not morphological cell differentiation, induced by withdrawal of LIF, was blocked by inhibition of p38 activity through PD169316 [63]. Duval *et al.*[64] compared the gene expression profiles of embryonic stem (ES) cell-derived differentiated cells by LIF withdrawal, in the presence or absence of PD169316. They showed that overexpression of MT-I prevented apoptosis of early differentiated cells [64]. These observations suggest that MT-I plays positive roles in the growth and anti-apoptosis of ES cells.

Maghdooni Bagheri *et al.*[65] examined the expression levels of MT among the hematopoietic precursor cell lines K562, DAMI, MEG-01 and EFL-153. They revealed that the more mature K562, DAMI and MEG-01 cells had higher MT levels than the immature ELF-153 cells. Treatment of K562 cells with phorbol ester leads to loss of their erythroid properties and to acquisition of several megakaryoblastoid charachteristics [66]. They further found that phorbol ester induction of K562 cells resulted in an increase in MT transcription and biosynthesis [65]. Consistently, the same group recently reported that in human cord blood cells, MT is more highly expressed in mature CD34^–^ cells than in immature CD34^+^ cells [67].

In the megakaryocytic lineage, it was recently reported that overexpression of MT-IIA in megakaryocytic DAMI cells caused increases in the cell size, intracellular granulation and levels of megakaryocytic-specific CD41 and CD42 with arrest of cell proliferation, suggesting a positive role for MT in megakaryocytic differentiation [68].

In the erythroid lineage, it has been reported that under conditions of induced erythropoiesis or anemia, the induction of MT in the rat bone marrow is enhanced, with predominant accumulation in erythroblasts [69]. However, in non-anemic rats, the induction of MT in bone marrow requires prior treatment with erythropoietin (EPO) [69]. The detection of an appreciable quantity of MT proteins in mature erythrocytes suggests that the source of MT in red blood cells may be induction of marrow erythroid precursor cells by EPO. Abdel-Mageed *et al.*[70] reported that EPO induced a three-fold increase in MT transcripts in K562 cells. MT induction was associated with EPO-induced cellular proliferation. However, EPO- or sodium butyrate-induced differentiation was inhibited in K562 cells stably transfected with an expression vector containing the human MT-IIA gene [70]. These findings may indicate a positive role for MT in the proliferation of erythroid cells, as well as a negative role for erythroid differentiation. Considering the positive role of MT for differentiation in the megakaryocytic lineage [68,70] and the negative role of MT for erythroid differentiation [68,70], the roles of MT toward differentiation may differ in different lineages.

Tsangaris *et al.*[71] examined the role of MTs in the apoptotic process by inhibiting their expression in the immature T cell line CCRF-CEM using antisense sequence-specific phosphorothioate oligodeoxynucleotides (ODNs). They found that the inhibition of MT synthesis by the ODNs stimulated the apoptotic process, as demonstrated by TUNEL assays. Collectively, the known experimental results for MT functions in hematopoietic cells are summarized in Table 1.

**Table 1:** Roles of MT in hematopoietic cells

**Lineage**	**Cells**	**Role**	**Ref.**
Undetermined	ES-derived differentiated cells	Overexpression of MT-I gene prevents LIF withdrawal induced apoptosis in ES derived differentiated cells	[64]
Undetermined	human cord blood cells	MT is more highly expressed in mature CD34^–^ cells than in immature CD34^+^ cells	[67]
Megakaryocyte	Megakaryocytic DAMI cells	Overexpression of MT-IIA resulted in the increase of cell size, intracellular granulation and increment of the megakaryocytic specific CD41 and CD42 and arresting in cell proliferation	[68]
Megakaryocyte, erythroid	K562, DAMI, MEG-01, ELF-153	The more mature K562, DAMI, and MEG-01 cell lines exhibited transcription of all MT isogenes, except MT-III and MT-IV.	[65]
		Phorbol ester induces increased MT transcription and biosynthesis.	
Erythroid	K562 cells	EPO- or sodium butyrate-induced differentiation as monitored by hemoglobin formation was inhibited in K562 cells stably transfected with an expression vector containing human MT-IIA gene.	[70]
Erythroid	Rat (acute blood loss, phenylhydrazine induced anemia model)	Induced erythropoiesis or anemia in rats, the induction of MT in the bone marrow is enhanced, with predominant accumulation in erythroblasts	[69]
T lymphoid	T cell line CCRF-CEM	Inhibition of hMTs synthesis by ODNs stimulated the apoptotic process	[71]

In the myeloid lineage, no studies analyzing MT functions have been published to date. Since MT possesses potent antioxidant functions and the generation of reactive oxygen species is important for the function of neutrophils for antibacterial activity, MT may also play a role in this lineage. Further clarification of the molecular functions of MT in this lineage may lead to better understanding of hematopoiesis.

### Expression analyses of MT in leukemia

Genetic mutations as well as overexpression of a certain gene play important roles in the pathogenesis of hematological malignancies [72]. In this context, alterations in the expression of MT genes have been reported in hematological malignancies (Table 2). Sauerbrey *et al.*[73] examined the expression of MT in initial (n = 92) and relapsed (n = 27) childhood acute lymphoid leukemia (ALL). Although there was a tendency for patients with initial ALL and MT expression to have a lower probability of disease-free survival than patients without MT expression, no differences in the percentages of MT positivity and staining intensity in children with either initial or relapsed ALL were noted [73]. They concluded that MT expression was independent of clinical prognostic factors such as age, sex, immunological subtype and initial blast cell count in patients with initial or relapsed ALL [73].

**Table 2:** Analyses of MT in hematological malignancies

**Type of diseases**	**Number of patients analyzed**	**Results of the analysis**	**Ref.**
ALL	119 (initial: n = 92, relapsed: n = 27)	Tendency in initial ALL with MT expression, the lower probability of disease free survival. However, no differences concerning MT percentage of positivity and intensity of staining in children with either initial or relapsed ALL. MT expression is not an important prognostic factor in the clinical drug resistance of childhood ALL.	[73]
ALL	47	After chemotherapy, MT positive cases (n = 18) showed maximal effect on the second day of treatment and apoptotic action completed on the tenth day. MT negative cases showed maximal effect on the first day of treatment and completed on the sixth day.	[23]
ALL	60	From microarray CGH analysis, BAK, CDKN2C, GSTM1 and MT-IF as a gene set that differed between ALL patients at diagnosis who had a risk or relapse from those who did not.	[24]
AML	19	The expression of resistance-related proteins P-170, GST-pi, Topo II, TS and MT was investigated. Patients who developed a relapse expressed more than two resistance mechanisms significantly more often than patients who remained remission (p = 0.005). The higher the number of resistance-related proteins in childhood AML the poorer the prognosis of the patients.	[25]
AML	43	mRNA expressions of the MT-IA, G and PU.1 genes were significantly, inversely correlated (MT-IG: R = −0.50, p < 0.001; MT-IA: R = −0.58, p < 0.0005).	[26]
HL	35	MT is differentially expressed in subclassified Hodgkin lymphoma. The number of MT-I, II immunostained cells is significantly higher in MCHL relative to other subclassified HL groups (p < 0.001), and also, the number of these cells is significantly higher in NSHL relative to NLPHL and LRCHL (p < 0.005).	[29]
DLBCL	115	MT labeling of more than 20% lymphoma cells was associated with a significantly poorer 5-year survival, independent of age, stage, or international prognostic index.	[27]
MPD	OMF (n = 9), CML (n = 11)	Increased GST-pi and MT expression in the bone marrow of MPD patients. Levels of MT in OMF patients were higher than in CML.	[28]

On the contrary, Tsangaris *et al.*[23] demonstrated that expression of MT was correlated with chemotherapy resistance. They examined MT expression in 47 children with ALL, and found that 18 cases were positive for MT expression at diagnosis. The mean apoptosis curve of these 18 cases showed that the maximal effect occurred on the second day of treatment, and the apoptotic action was completed on the tenth day. In contrast, the mean apoptosis curve of the 29 MT-negative cases revealed higher sensitivity to the treatment, with the maximal apoptotic effect on the first day and completion of the apoptotic action on the sixth day. A recent study by Usvasalo *et al.*[24] using microarray comparative genomic hybridization (CGH) analyses revealed a prognostic classifier in adolescent and young adult ALL patients aged 10–25 years (n = 60). They demonstrated that BCL2-antagonist/killer 1 (BAK1), cyclin-dependent kinase inhibitor 2 C (CDKN2C), glutathione S-transferase M 1 (GSTM1) and MT-IF formed a gene set that differed between ALL patients at diagnosis who had a risk of relapse and those who did not [24].

In AML, the expression of the resistance-related proteins P-glycoprotein 170 (P-170), glutathione-S-transferase pi (GST-Pi), topoisomerase-II (Topo II), thymidylate synthase (TS) and MT was investigated in leukemic cells from 19 children with newly diagnosed AML [25]. Different percentages of positivity for the examined proteins were noted. MT was expressed in leukemic cells from 68% of cases with newly diagnosed AML. Although the number of patients was small, they concluded that patients who developed relapse showed a poorer prognosis, and frequently expressed more than two resistance-related proteins, including MT, compared with patients who remained in remission [25].

The author’s research group recently revealed that the mRNA expressions of the MT-IA and MT-IG genes were significantly inversely with the PU.1 gene correlated in 43 primary acute AML specimens (MT-IG: R = −0.50, p < 0.001; MT-IA: R = −0.58, p < 0.0005) [26]. Although the clinical outcomes of these patients were not analyzed, we demonstrated that these genes were directly regulated by the epigenetic activities of the hematopoietic transcription factor PU.1. In 24 AML cases, we previously reported that PU.1 expression was inversely correlated with the tyrosine kinase receptor FLT3 [74], and that strong expression of wild-type FLT3 was an unfavorable prognostic factor for overall survival [75,76]. In addition, PU.1 expression was reported to be a positive indicator for other hematological malignancies, such as follicular lymphoma [77]. Although further extensive analyses are required, it is possible that increased MT-I expression represents a poor prognostic marker for AML.

### Expression analyses of MT in lymphoma and myeloproliferative disorders

In lymphoid malignancies, it has been indicated that the MT-I and MT-II expression profiles may be useful for discriminating between non-malignant and malignant lymphoid neoplasms [27,78]. Hodgkin lymphoma (HL) is a neoplastic disease consisting of two distinct entities: nodular lymphocyte predominant HL (NLPHL) (5%) and classical HL (CHL). CHL includes four histological subtypes: nodular sclerosis HL (NSHL), mixed cellularity HL (MCHL), lymphocyte-rich classical HL (LRCHL) and lymphocyte depleted classical HL (LDHL) [79]. Frequencies of these subtypes are, NSHL: 70%, MCHL: 20-25%, LRCHL: 5%, LDHL : <1% [79]. Among these, LRCHL has most favorable prognosis, less frequently associated with B symptoms, bulky disease and mediastinal involvolvement compared with MCHL and NSHL, and almost half of the patients present in stage I. Penkowa *et al.*[29] analyzed MT expression in the lymph nodes of 34 patients with HL, comprising 15 patients with NSHL, 11 patients with MCHL, five patients with LRCHL and three patients with nodular lymphocyte-predominant HL (NLPHL), and in controls. NSHL and MCHL patients showed highly increased MT expression throughout the lymph nodes. In contrast, MT expression was barely increased in LRCHL patients relative to the controls. NLPHL patients showed a distinct pattern of heterogeneous MT expression, with increased MT in nodular areas surrounded by MT-negative tissues. Therefore, MT is differentially expressed in HL subclassifications [29]. However, they also revealed that there were no correlations between the clinical outcomes after chemotherapy and the numbers of MT-I- and MT-II-positive cells in the lymph node parenchyma of the patients with HL.

However, in diffuse large B-cell lymphomas (DLBCLs), a significant inverse correlation between MT expression and clinical outcome has been reported [27]. Through mRNA profiling using Affymetrix assays, Poulsen *et al.*[27] demonstrated that MT mRNA was upregulated in 15 of 48 DLBCLs, including 12 of 23 activated B-cell type lesions and three of nine type-3 lesions. In contrast, MT mRNA was low to undetectable in 16 germinal center B-cell type DLBCLs. Only one of 15 patients with upregulated MT mRNA achieved sustained remission, suggesting that upregulated MT mRNA constitutes a significant risk factor for treatment failure. Importantly, they expanded their immunohistochemical analysis to include an additional 67 DLBCLs (total, 115 DLBCLs), and found that MT labeling of >20% of lymphoma cells was associated with a significantly (p = 0.004) poorer 5-year survival, independent of age, stage or international prognostic index [27].

Wrobel *et al.*[28] analyzed the expressions of MT and GST-Pi in bone marrow of patients with myeloproliferative disease (MPD). Although the number of patients was small, comprising nine patients with osteomyelofibrosis (OMF) and 11 patients with chronic myelocytic leukemia (CML), they revealed increased GST-Pi and MT expression in the bone marrow of MPD patients. The levels of MT expression in the OMF patients were higher than those in the CML patients. These findings suggest that MT expression may be correlated with bone marrow fibrosis.

## Conclusions

Increasing evidence suggests that MT plays important roles in oncogenesis through multiple functions. MT functions, such as those in cell survival, cell growth, drug resistance and differentiation have been precisely studied in non-hematological diseases. To date, more than 9,700 papers have been published about this multifunctional protein related to tumorigenesis, toxicology, cancer chemotherapy, inflammation, aging, and so on. In this review, the author focused on MT functions in hematopoietic cells, and the roles of MT in these cells that have been described. Collectively, in these cells, MT has been reported to play roles in hematopoietic cell differentiation [64,65,67-69], hematopoietic cell proliferation [64,70] and prevention of apoptosis [71] (Table 1). It may also be related to drug resistance in AML [25] and ALL [23], and is a poor prognostic factor in DLBCL [27] (Table 2). Expansion of these directions toward hematological malignancies may further clarify the mechanisms underlying hematological malignancies.

## Competing interests

The author declares no competing interests.
